# Faulty Administration from Ignorance of Modern Hospital Progress

**Published:** 1914-11-07

**Authors:** Henry Burdett


### REPORTS ON
### Hospitals of the United Kingdom.

### FAULTY ADMINISTRATION FROM IGNORANCE OF MODERN HOSPITAL PROGRESS.

By SIR HENRY BURDETT, K.C.B., K.C.V.O.

We have now arrived at a date when the inspections of the principal voluntary hospitals in England and Wales upon which we have been engaged since the autumn of 1909 are almost completed. These inspections, with the criticisms, reconstructions, changes in administration, and extended and commendable efforts on the part of the managers of our voluntary hospitals throughout the country to bring their institutions up to date, will combine to secure widespread interest for the Reports, and all that has arisen out of them, when republished in a separate volume, owing to the practical value of its contents. At the outset we were urged by important organs of public opinion to report fearlessly what we found, and to spare no effort to raise the standard of the institutions inspected, so far as possible, to one of uniform efficiency. This arduous and responsible task we have endeavoured to fulfil, but most of the success secured must stand to the credit of the responsible managers, to whose co-operation and hearty interest the ensuing results of real importance to the institutions concerned are largely due.

It has been our endeavour by a personal inspection of the principal hospitals and Poor-Law infirmaries throughout England and Wales, and by frankly outspoken Reports, to enable the managers of these institutions to know exactly where they stand, where improvements could be introduced, and so to attain a uniformly high standard of hospital efficiency. It was undoubtedly a bold step to take for one man to make these inspections and to embody their results in critical Reports with the aim of publishing them in a volume which would record the condition of the English voluntary hospitals and larger Poor-Law infirmaries in the first decade of the twentieth century. It is a matter of genuine satisfaction and thankfulness that these Reports have proved welcome and helpful to many zealous workers connected with voluntary hospitals throughout the United Kingdom. With all such workers we are united by the spirit which has animated our work for the voluntary hospitals, for every one of us believes in the doctrine of personal service in the days of health in the cause of the sick.

Readers of The Hospital will have in remembrance the Report which we published on the Cumberland Infirmary, Carlisle, which appeared in The Hospital of September 19 last. Out of that Report a correspondence has arisen in which Mr. Lediard, F.R.C.S., senior surgeon, has taken an active part. From this correspondence it is made fairly clear that Dr. Lediard has not been wholly in sympathy with the important scheme of reconstruction recently carried out at the instance of Dr. Henry Barnes, the consulting physician, and his friends at a cost in round figures of £30,000. Possibly two parties may have arisen amongst the Governors, but of this we have no knowledge. In any case, the letter published in the *Lancet* of October 31 from Dr. Lediard has a special interest because it reveals an ignorance of the standards of a modern hospital, and of the essential necessity in the present day, when the cost of working our voluntary hospitals has so materially increased, for the administration in every department to be thoroughly up to date. Where this is not so, there financial stress and other troubles must arise which may prove fatal to efficiency. It must further prevent an institution from attaining the maximum of usefulness which its work ought to secure for the population in the district which it is privileged to serve. The best and simplest way of illustrating the force which attaches to this criticism seems to us to be to republish Dr. Lediard's letter from the *Lancet* and to deal with it paragraph by paragraph. We have numbered the paragraphs into which Dr. Lediard's letter is divided and print them in italics. Our comments follow each paragraph in ordinary type:—

### The Accommodation Provided.

(1) *Sir Henry Burdett is correct in stating that on the cover of the report of the Cumberland Infirmary for 1913 there may be found a slip of paper which declares "there is accommodation for 150 in-patients," but owing to lack of funds only 102 beds are authorised for occupation. It is not, therefore, incorrect to describe our hospital as a small provincial institution. We could not put up more than 130 patients at most (unless on balconies or open-air spaces), even if our finances permitted. Thirty beds in the disused portion of the infirmary have recently been inspected by the War Office, but only twenty have been considered fit for occupation by wounded or sick.*

It will be noted that Dr. Lediard questions the accuracy of there being accommodation for 150 beds, for he declares "we could not put up more than 130 patients at most," and the War Office, after inspection, have found that only twenty of the extra thirty beds would be fit for occupation by wounded or sick. If this be so, it is remarkable that £30,000 should have been spent upon the reconstruction, as the cost, considered from this aspect, must have been high indeed. Still, 120 beds is a goodly number, and, having regard to the average size of some excellent up-to-date provincial hospitals, the Cumberland Infirmary cannot justly be described as a small provincial institution.

A Faithful Record of the Condition of the Hospitals Inspected.

(2) *Sir Henry Burdett tells us that his report on our infirmary is intended to be republished, with some hundred others, as a faithful record of the condition in which he found it in June 1914. His visit lasted at most two hours, and I cannot regard his report as in any sense a faithful record. In the visitors' book of the infirmary over his signature I find the following:—*

*"It has been a pleasure to inspect this hospital after twenty-one years. Since 1892 much has been done to improve the hospital and to add to its efficiency. The working and planning of the new portions of the hospital would be greatly aided if the ward units were made complete by the addition of a suitable airy temporary larder to each ward where the food can be kept, by the provision of a housemaid's cupboard, of patients' clothes cupboards, and other details which are now included in modern hospital construction. The accommodation for the nurses is attractive and good. That for the domestic staff a great improvement on what formerly prevailed, but I should like to see one good room comfortably furnished as a sitting room for the members of this staff."*

*When Sir Henry Burdett's faithful record appears will he include this impression? Or will he republish the following remarks from* The Hospital:—

*"Administratively, however, it seemed to us that it has been changed sadly for the worse. . . . It was a sad disappointment to find an absence of the old spirit and atmosphere of twenty years before. . . . We are convinced something is radically wrong at this institution."*

*Satisfied as Sir Henry Burdett appears to have been after his inspection of the Cumberland Infirmary in June 1914, some unexplained change came over him.*

Dr. Lediard raises three points in these paragraphs: (*a*) A two hours' inspection cannot produce a report containing a faithful record. As to this, we have to point out that forty-five years' continuous work and close association with the administration of the voluntary hospitals, and indeed of all kinds of hospitals, on the part of an inspector simplifies his work, and two hours may be more than ample to complete a full inspection of an institution containing 102 beds for the reception of patients. It is not the length of time but the knowledge and close familiarity with every detail of the working of a hospital which secures a faithful report by the inspector.

(*b*) The Report contained a detailed endorsement of the good points noticeable in the hospital, some of which, though not precisely produced in the Report published, are recorded by the inspector in the visitors' book of the hospital, which is open to the inspection of visitors, Governors, and all interested in the institution. When the inspector's Report further states that the administration has been changed sadly for the worse, and that something is radically wrong at the institution, the two statements are declared to be conflicting and to indicate unfaithfulness on the part of the inspector who is responsible for both. Such a contention shows a surprising disregard for the proprieties on the part of Dr. Lediard, seeing that the object of the inspector is to promote the increased efficiency of each hospital inspected and to help it in every possible way. Criticism of the administration, though it may constitute a necessary portion of a faithful report, would be unjustifiable and out of place in the visitors' book, which lies open to general inspection, as just indicated. So far from being conflicting, therefore, the character and existence of the two statements prove the faithfulness of the record, as every knowledgeable hospital manager will agree.

(*c*) Dr. Lediard states that the existence of the two statements proves that some unexplained change came over the inspector subsequent to his leaving the infirmary. Dr. Lediard is entitled to hold any opinion he chooses, but the insinuation that some outside influence was brought to bear on the inspector between the date of his leaving the infirmary and the writing of his Report has no foundation in fact. The absurdity of prejudice in this connection is amusingly proved by another critic of the Report, who claimed that the influence suggested was exercised upon the inspector prior to his visit to Carlisle!

Reverence for the Dead and Consideration for the Living.

(3) *Sir Henry Burdett has condemned our mortuary as having been "erected on a defective plan," and says that every properly organised voluntary hospital has a mortuary chapel or view room, so that "reverence for the dead and due consideration for the living may always be secured." The mortuary in the Cumberland Infirmary was built in 1903, together with a post-mortem room, at a cost of £500, and stands by itself at some distance from any ward. The mortuary or "view room" resembles a small chapel, having an open-timbered roof, a red-tiled floor, with leaded and coloured glass windows at each end. In it four low beds, supplied with fine hemstitched linen, can be found, and a little altar table upon which rest a brass cross and vases for flowers placed beneath one window and a "prie Dieu" below the other window. In this "view room" I maintain that all due "reverence for the dead and due consideration for the living" can be secured.*

A view room is a mortuary chapel containing one bier only, where the friends can take their last farewell of their dear ones. A chamber containing four low beds, each of which may contain a dead body, despite the hemstitched linen and other adjuncts, cannot and does not display the reverence for the dead and the due consideration for the living which every up-to-date hospital ought to secure as a matter of education and sentiment.

"Finances not in a Prosperous State."

(4) *It seems that Sir Henry Burdett cannot understand that in our one clinical room we have*

*all that we require at present and our existing medical staff could not conveniently utilise more than one such room. It may be pointed out to Sir Henry Burdett that if our finances were in a prosperous state we should like to provide an x-ray room of three times the size of the one we now have, and that we have no room set apart for the honorary dental surgeon. These and other defects could be remedied, not so much by the possession of the "old spirit and atmosphere" (as Sir Henry Burdett might think) as by the acquisition of increased funds.*

All will agree that the clinical room is an essential of the ward unit of every up-to-date hospital where the treatment and medical work are modern and efficient.

Has Dr. Lediard ever asked himself why the finances of the Cumberland Infirmary are not in a prosperous state? If he knew as much about the raising of money for voluntary hospitals as some of us do, he would recognise that the state of the finances at Carlisle, taken as they stand, coupled with the fact that the new extensions, if they remain unoccupied, must speedily deteriorate, strongly enforces the inspector's recommendation that a special investigation and independent report by a hospital expert of wide experience into the whole question of the present character of the administrative arrangements should be ordered and presented to the Governors without delay.

We have ventured to point out the misapprehensions which permeate Dr. Lediard's latest contribution, because they must prove of interest to hospital managers, for they testify to the causes which frequently underlie defects in administration, and the surprising difficulty which is sometimes experienced to secure a full investigation and the speedy adoption of a new and better system of administration. We all have to live and learn, and we hope that Dr. Lediard and the Governors of the Cumberland Infirmary who may agree with him will reconsider the whole matter, in a friendly spirit, from the point of view of the best interests of their institution and of the population it was established to provide for in the day of sickness.

